# Evaluation of quality of life in patients with schizophrenia: An inpatient social welfare institution-based cross-sectional study

**DOI:** 10.1515/med-2024-0947

**Published:** 2024-04-06

**Authors:** Aleksandra D. Petrovic, Ana M. Barjaktarevic, Olivera Z. Kostic, Jelena M. Dimitrijevic, Sara S. Mijailovic, Andjela D. Gogic, Slobodan M. Jankovic, Marija V. Andjelkovic, Marijana S. Stanojevic Pirkovic, Katarina D. Parezanovic Ilic, Marina J. Kostic, Vladimir S. Janjic

**Affiliations:** Department of Pharmacology and Toxicology, Faculty of Medical Sciences, University of Kragujevac, Kragujevac, Serbia; Department of Pharmacy, Faculty of Medical Sciences, University of Kragujevac, Kragujevac, Serbia; Center for Research on Harmful Effects of Biological and Chemical Hazards, University of Kragujevac, Kragujevac, Serbia; Department of Medical Statistics and Informatics, Faculty of Medical Sciences, University of Kragujevac, Kragujevac, Serbia; Department of Medical Biochemistry, Faculty of Medical Sciences, University of Kragujevac, Kragujevac, Serbia; Department of Physical Medicine and Rehabilitation, Faculty of Medical Sciences, University of Kragujevac, Kragujevac, Serbia; Department of Psychiatry, Faculty of Medical Sciences, University of Kragujevac, Kragujevac, Serbia

**Keywords:** quality of life, schizophrenia, questionnaires, social welfare institution

## Abstract

Schizophrenia is a chronic mental illness with a poor quality of life (QoL). The main aim of this study was to measure the QoL and factors that affect the QoL of patients with schizophrenia placed in a social welfare institution. This cross-sectional study included 287 patients with schizophrenia who were treated in a long-stay social care institution in which QoL was assessed using five different instruments: the World Health Organization Quality of Life scale, the EuroQoL Five-Dimension-Five-Level scale (including the visual analog scale), the Quality of Life Enjoyment and Satisfaction Questionnaire – Short Form, and the Brief Psychiatric Rating Scale. To determine the impact of patients’ characteristics on score values, multiple linear regression using backward elimination was employed. Due to non-normality in the distribution of the dependent variables, a Box–Cox power transformation was applied to each dependent variable prior to conducting multiple linear regression analysis. Results revealed that patients with schizophrenia have lower QoL. Our study revealed that age, level of education, type of accommodation, type of pavilion, age of onset of the disease, number of prescribed antipsychotics, number of psychiatric comorbidities, duration of therapy, and the number of daily doses of antipsychotics are dominant contributors to the QoL in patients with schizophrenia who were treated in social welfare institution.

## 1 Introduction

Schizophrenia is one of the most severe, chronic mental illnesses that significantly disables patients, affects all important areas of their lives, and has a negative impact on the quality of life (QoL) [1]. It is characterized by numerous positive (hallucinations, delusions), negative (social withdrawal, lack of emotions), and cognitive symptoms (attention deficit disorder), with a different etiology and a prevalence of approximately 1% of the world’s population [2]. The total costs are disproportionate to the prevalence of the disease because the disease itself represents a significant economic burden for patients, society, and the health system, and the World Health Organization ranks schizophrenia among the top ten diseases that burden the global health system [3,4]. Schizophrenia outcomes have not improved despite vastly expanded knowledge about the etiology, pathophysiology, and course of schizophrenia, and changes in the concept of treatment are mostly related to the individual’s perception of their own QoL [5,6].

Patients diagnosed with schizophrenia suffer from various disorders, such as personality disorders, social dysfunction, associated comorbidities, emotional instability, and physical inactivity, all of which together lead to diminished QoL [7]. Institutions, where such patients are treated, can be categorized as either short-stay or long-stay inpatient facilities, depending on factors such as illness severity, duration, family circumstances, functional ability, social adjustment, level of self-care, and the need for external care [8,9]. In certain countries, patients who stay in these institutions for a period longer than 6 months receive the status of new users of social and healthcare services in a long-stay inpatient facility [10]. In this type of institution, patients receive proper treatment and care, and rehabilitation, which gives them increased security and a better QoL [11]. As opposed to this, staying at home and being treated in primary health care are not significant factors in enhancing the QoL for these patients [12]. Assessing the QoL and identifying influential factors has become an essential component of patient care, with particular attention given to vulnerable groups such as schizophrenia patients placed in social welfare institutions [13].

The aim of this research was to assess the QoL of patients diagnosed with schizophrenia and factors that determine it in patients who were treated in a social welfare institution. The assessment was conducted using the following scales: the World Health Organization Quality of Life scale (WHOQOL-BREF), the EuroQoL Five-Dimension-Five-Level scale (EQ-5D-5L) including the EQ-VAS (visual analog scale), the Quality of Life Enjoyment and Satisfaction Questionnaire – Short Form (Q-LES-Q-SF), and the Brief Psychiatric Rating Scale (BPRS).

## 2 Methods

The study was designed as a cross-sectional study, and it was conducted in a social welfare institution in Central Serbia. This inpatient social welfare institution provides care for approximately 890 patients from across Serbia who have been experiencing long-term psychiatric disorders.

A total of 287 patients diagnosed with schizophrenia (F20.0–F20.9), according to the tenth International Statistical Classification of Diseases and Related Health Problems, participated in the research [14]. The inclusion criteria consisted of preserved reasoning ability related to the research, established diagnosis of schizophrenia, users aged 18 or older, and a minimum length of stay in the institution of 12 months. Exclusion criteria encompassed conditions such as dementia, autism, mental retardation, cognitive disorders that hinder understanding and responding to questionnaire questions, illiteracy of users, and visual impairments preventing reading the questionnaire. The sample also included the results of 153 patients from a previous pilot study conducted by the authors [15].

To estimate the QoL, we used the following scales: WHOQOL-BREF, EQ-5D-5L including the EQ-VAS, Q-LES-Q-SF and the rating scale for measuring psychiatric symptoms (BPRS). The study was performed in the social welfare institution where patients with schizophrenia were treated encompassing the first, second, and third pavilions of this institution in accordance with the patient’s daily obligations and needs. Data collection was performed during the following time period, from April 1 to September 1, 2023. The data were collected by the first author and multi-disciplinary team of social welfare institution including psychiatrists, occupational therapists, psychologists, general physicians, and nurses who were involved in the treatment of the study population. All patients who satisfied the inclusion criteria provided their answers regarding the scales mentioned above. The first author of the study with the help of medical workers conducted an interview with each patient individually and according to the patients’ responses and behaviors during the interview the first author completed the scales in collaboration with medical staff.

Questionnaires pertaining to QoL were translated and adapted according to international standards: double translation from English to Serbian, harmonization of the translation, reversed translation to English, harmonization of the final translation, and pilot testing involving a group of eight respondents. For each scale for this research, permission was obtained from the author of the scale and other copyright holders, or the questionnaires were downloaded from the official websites of the respective scales. The reliability of the questionnaire was examined by forming a correlation matrix and calculating Cronbach’s alpha. Criterion validation was performed by comparing each questionnaire with previously translated WHOQOL and EQ-5D questionnaires and conducting correlation calculations. Additionally, content validation was conducted by a panel of psychiatrists [15,16].

The answers that patients provided were interpreted in accordance with the instructions from the authors of the mentioned questionnaires. Regarding the WHOQOL-BREF, the score values of each question are placed in a positive direction where a higher score corresponds to better values of QoL. Concerning the EQ-5D-5L, the unique state of health was converted into individual index values where high index values correspond to a better health condition of the patient, ranging from 0.0 to 1.0. The visual analog scale (VAS) is the part of EQ-5D-scale and has values in a range from 0 to 100, indicating the current state of health which is based on the personal perception of the patients, where high score values correspond to a better QoL of patients and *vice versa*. Similarly, higher values of Q-LES-Q-SF questionnaire indicate better QoL and life satisfaction in patients, while lower values have opposite meaning within the range of the corrected raw score in percentage with values from 0 to 100. Considering the values of BPRS, the minimum possible raw score of value 24 indicates the absence or minimal severity of psychiatric symptoms and better QoL, and on the other hand, the maximum value of 168 indicates severe psychiatric symptoms and low QoL. [15,16].

For the purposes of this research, we collected the following sociodemographic characteristics and clinical data from the users and from medical documentation: gender, age, education, year of disease onset, length of stay in the institution, diagnosis, presence of general comorbidities, psychiatric comorbidities, group of prescribed antipsychotics, type of antipsychotic, number of antipsychotics per user, daily dosage of antipsychotics, prescribed dose of antipsychotics, and duration of therapy. From the patient interviews, the following variables were determined: smoking status (whether they are smokers or not), coffee consumption (whether they drink coffee or not), type of pavilion where the pavilions were designated with numbers 1, 2, and 3, with a higher number indicating a worse functional status of the patients, characterized by poorer mobility and a greater need for daily assistance (first, second, or third pavilion), and type of accommodation (four-bed, five-bed, or six-bedroom). Doses of antipsychotics were expressed based on chlorpromazine equivalent doses (CPZ equivalent) [17].

### 2.1 Statistical analysis

For performing statistical analysis, the SPSS program version 22 (IBM SPSS Statistics 22, Armonk, NY, USA) was used. All continuous variables measured in this study are presented as either arithmetic mean and standard deviation or the median and interquartile range (IQR). Categorical variables are presented as the number and frequency for each category. The normality of the distribution was examined by using the Kolmogorov–Smirnov test. Multiple linear regression using backward elimination was employed to determine the effect of patients’ characteristics on score values. Since the dependent variables in the regression model did not meet the assumption of normality, a Box–Cox power transformation was performed for each of them before conducting multiple linear regression. The significance level for all statistical tests was set at 0.05.


**Ethical approval:** This research has been complied with all the relevant national regulations, institutional policies, and in accordance with the tenets of the Helsinki Declaration and has been approved by the competent Ethical Committee of social welfare institution and the Ministry of Labor and Social Policy, Department for Family Care and Social Protection (Ethical Committee decision number: 01-1324/2).
**Informed consent:** Informed consent has been obtained from all individuals included in this study.

## 3 Results

A total of 287 patients diagnosed with schizophrenia participated in this research. Among them, 162 (56.4%) were men, and 125 (43.6%) were women. The average age of the patients was 52.03 ± 10.522, with the youngest patient being 20 years old and the oldest being 81 years old. The majority of patients had a high school degree (*N* = 135 or 47.0%), followed by those with an elementary school degree (*N* = 95 or 33.1%), a university degree (*N* = 33 or 11.5%), and who had not completed elementary school (*N* = 24 or 8.4%). Table 1 presents the disease characteristics of the patients, along with the arithmetic mean, median, and IQR values.

**Table 1:** The disease characteristics of the patients

Patient characteristics	Arithmetic mean ± standard deviation	Median (IQR)
Length of stay in the institution (years)	11.86 ± 9.593	10.0 (16)
Year of disease onset	29.25 ± 10.582	28.0 (18)
Length of the prescribed therapy (months)	12.51 ± 11.471	8.0 (18)
Chlorpromazine equivalent	397.63 ± 289.71	306.25 (300)
Number of general comorbidities	1.38 ± 1.040	1.0 (1)
Number of psychiatric comorbidities	0.65 ± 0.816	0.0 (1)
Number of all comorbidities (general and psychiatric)	2.03 ± 1.344	2.0 (2)

**Patient characteristics**	**Number (Percent)**	**Chi-square test/** ***p***
**Type of schizophrenia**		
Residual schizophrenia	47 (16.4)	12.355/0.015
Paranoid schizophrenia	62 (21.6)	
Hebephrenic schizophrenia	40 (13.9)	
Unspecified schizophrenia	71 (24.7)	
Simple schizophrenia	67 (23.3)	
**General comorbidities**		
No	73 (25.4)	110.098/0.000
Obesity	27 (9.4)	
Bronchial asthma	17 (5.9)	
Hypertension	61 (21.3)	
Hypertension/bronchial asthma	27 (9.4)	
Hypertension/diabetes	22 (7.7)	
Hypertension/diabetes/thyrotoxicosis	14 (4.9)	
Benign prostate hyperplasia	15 (5.2)	
Hypertension/diabetes/obesity	31 (10.8)	
**Psychiatric comorbidities**		
No comorbidities	162 (54.4)	262.251/0.000
Depression	63 (22.0)	
Depression/personality disorder	25 (8.7)	
Depression/alcoholism	17 (5.9)	
Depression/Parkinson’s disease	20 (7.0)	
**Group of prescribed antipsychotics**		
Typical	23 (8.0)	94.836/0.000
Atypical	156 (54.4)	
Combination	108 (37.6)	
**Type of prescribed antipsychotics**		
Risperidone/haloperidol	16 (5.6)	147.718/0.000
Risperidone	77 (26.8)	
Clozapine	28 (9.8)	
Olanzapine	31 (10.8)	
Haloperidol	19 (6.6)	
Risperidone/olanzapine	12 (4.2)	
Clozapine/olanzapine	8 (2.8)	
Clozapine/olanzapine/haloperidol	17 (5.9)	
Olanzapine/haloperidol	15 (5.2)	
Clozapine/haloperidol	18 (6.3)	
Risperidone/clozapine	24 (8.4)	
Aripiprazole	22 (7.7)	
**Daily dosage**		
Once daily	48 (16.7)	35.631/0.000
Twice daily	119 (41.5)	
Thrice daily	120 (41.8)	
**Number of prescribed antipsychotics**		
One	174 (60.6)	128.829/0.000
Two	96 (33.4)	
Three	17 (5.9)	

Out of the total number of patients, 253 (88.2%) of them reported consuming coffee, while 209 (72.8%) of them reported being smokers. Regarding the type of accommodation, 12 (4.2%) patients stayed in rooms with 4 beds, 213 (74.3%) in rooms with 5 beds, and 62 (21.6%) in rooms with 6 beds. In addition, a total of 130 (45.3%) patients were accommodated in the first pavilion, 94 (32.8%) in the second pavilion, and 63 (22.0%) patients in the third pavilion.

Patients in this study had an average EQ-5D-5L score of 0.824 ± 0.167 out of a maximum of 1.0, while the average VAS score was 78.415 ± 15.756 out of a maximum of 100. Regarding the Q-LES-Q-SF score, patients had an average of 65.997 ± 13.796, with the maximum possible value being 100. On the BPRS, the maximum possible value was 168, while the average value in this study was 36.885 ± 12.134. Table 2. presents the mean and standard deviation of the scores of all examined scales for the entire population as well as their median and IQR values. Multiple linear regression models obtained by Backward elimination methods showed that the EQ-5D-5L score was statistically significantly related to age, while age and type of accommodation showed a statistically significant influence on VAS score. Daily dosages, tobacco consumption, coffee consumption, and type of pavilion were factors that had a statistically significant effect on BPRS score values. The summary presentation of the factors with the most significant influence on each examined score is presented in Table 3. Analysis revealed strong positive correlations among the QoL measures, indicating a cohesive understanding of well-being across the instruments. The negative correlation observed between BPRS scores and QoL assessments suggests that higher psychiatric symptom severity is associated with lower perceived QoL, supporting the construct validity of the measures (Table 4).

**Table 2:** The quality of life assessed using EQ-5D-5L, VAS, Q-LES-Q-SF, BPRS, and WHOQOL-BREF

Scale	Arithmetic mean ± standard deviation	Median (IQR)
EQ-5D-5L score	0.824 ± 0.167	0.848 (0.292)
VAS score	78.415 ± 15.756	80.0 (20.0)
Q-LES-Q-SF score	65.997 ± 13.796	66.0 (14.0)
BPRS score	36.885 ± 12.134	33.0 (15.0)
WHOQOL-BREF physical health domain	67.429 ± 12.843	69.0 (19.0)
WHOQOL-BREF psychological domain	66.540 ± 13.048	69.0 (19.0)
WHOQOL-BREF social relationships domain	60.324 ± 17.039	56.0 (25.0)
WHOQOL-BREF environment domain	70.916 ± 13.719	69.0 (18.0)

**Table 3:** Factors with the most significant influence on each examined score

	*B*	Beta	*p*	Confidence Interval
**EQ-5D-5L score**				
Transformation *Y* ^λ^ (*λ* = 1.86); *F* = 17.644, *p* = 0.000, *R* ^2^ = 22.5%				
Age	−0.003	−0.139	**0.009**	−0.006 to −0.001
**VAS score**				
Transformation *Y* ^λ^ (*λ* = 1.7); *F* = 13.325, *p* = 0.000, *R* ^2^ = 13.2%				
Age	−7.515	−0.143	**0.014**	−13.510 to −1.520
Type of accommodation	132.641	0.115	**0.042**	4.622 to 260.659
**Q-LES-Q-SF score**				
Transformation *Y* ^λ^ (*λ* = 1.43); *F* = 17.644, *p* = 0.000, *R* ^2^ = 22.5%				
Length of stay in the institution (years)	−0.236	−0.164	**0.004**	−0.398 to −1.091
**BPRS score**				
Transformation *Y* ^λ^ (*λ* = 1.16); *F* = 42.625, *p* = 0.000, *R* ^2^ = 53.8%				
Daily dosage	−0.085	−0.141	**0.003**	−0.140 to −0.030
Tobacco consumption	−0.097	−0.098	**0.024**	−0.181 to −0.013
Coffee consumption	0.106	0.104	**0.015**	0.021 to 0.191
Type of pavilion	0.049	0.087	**0.040**	0.002 to 0.095
**WHOQOL-BREF physical health domain**				
Transformation *Y* ^λ^ (*λ* = 1.26); *F* = 14.188, *p* = 0.000, *R* ^2^ = 18.7%				
Age	−0.478	−0.105	**0.050**	−0.962 to −0.006
Type of pavilion	11.011	0.181	**0.001**	4.439 to 17.584
**WHOQOL-BREF psychological domain**				
Transformation *Y* ^λ^ (*λ* = 1.65); *F* = 28.413, *p* = 0.000, *R* = 16.1%				
Number of psychiatric comorbidities	−42.683	−0.344	**0.000**	−27.381
**WHOQOL-BREF social relationships domain**				
Transformation *Y* ^λ^ (*λ* = 0.96); *F* = 6.370, *p* = 0.000, *R* = 11.7%				
Year of disease onset	−0.227	−0.177	**0.002**	−0.372 to −0.083
Length of stay in the institution (years)	−0.269	−0.189	**0.001**	−0.433 to −0.105
Number of prescribed antipsychotics	−3.341	−0.149	**0.011**	−5.907 to −0.774
Length of the prescribed therapy (months)	0.172	0.145	**0.011**	0.039 to 0.305
Number of psychiatric comorbidities	1.973	0.118	**0.037**	0.116 to 3.830
**WHOQOL-BREF environment domain**				
Transformation *Y* ^λ^ (*λ* = 1.64); 9.1% of variance explained; *F* = 4.584, *p* = 0.000, *R* = 9.1%				
Level of education	−57.444	−0.138	**0.019**	−105.207 to −9.680
Length of stay in the institution (years)	−5.619	−0.162	**0.006**	−9.651 to −1.587
Group of prescribed antipsychotics	−80.723	−0.148	**0.022**	−149.955 to −11.492
Daily dosage	57.709	−0.126	**0.049**	0.227 to 115.190
Type of accommodation	129.929	0.186	**0.001**	50.426 to 209.613

The bold values represent statistically significant results, as determined by the application of a significance threshold of *p* < 0.05.

**Table 4:** Correlation matrix

Total score values	EQ-5D-5L score	VAS score	Q-LES-Q-SF score	BPRS score	WHOQOL-BREF physical health domain	WHOQOL-BREF psychological domain	WHOQOL-BREF social relationships domain	WHOQOL-BREF environment domain
EQ-5D-5L score	**1**							
VAS score	**0.437**	**1**						
Q-LES-Q-SF score	**0.519**	**0.460**	**1**					
BPRS score	−**0.348**	−**0.196**	−**0.209**	**1**				
WHOQOL-BREF physical health domain	**0.478**	**0.352**	**0.567**	−**0.265**	**1**			
WHOQOL-BREF phycological domain	**0.527**	**0.484**	**0.605**	−**0.345**	**0.620**	**1**		
WHOQOL-BREF social relationships domain	**0.248**	**0.306**	**0.559**	−0.093	**0.396**	**0.439**	**1**	
WHOQOL-BREF environment domain	**0.252**	**0.266**	**0.522**	−0.098	**0.401**	**0.452**	**0.495**	**1**

The bold values represent statistically significant results, as determined by the application of a significance threshold of *p* < 0.05.

## 4 Discussion

In our study, we examined the perspective of QoL in patients with schizophrenia who are placed in an inpatient institution. This assessment encompassed various domains, including subjective feelings toward the applied therapy. In recent decades, there has been significant attention given by researchers to investigate the QoL among individuals with mental disorders. The experience of QoL in patients serves as an important indicator of treatment success in schizophrenia, and the instruments used to measure it possess different psychometric, social, and therapeutic characteristics [18,19]. The simultaneous application of several questionnaires within the same population, as done in our research, is increasingly prevalent in modern psychiatric practice.

The results of our research revealed that elderly patients have lower values in EQ-5D-5L scores, VAS scores, and scores for the physical health domain of the WHOQOL. These results are to be expected, as advancing age is associated with significant physiological changes that negatively impact overall health, daily physical functioning, and self-care abilities. Consequently, there is a greater reliance on daily medical assistance, particularly within the group of patients with psychiatric conditions. Our results are in compliance with similar studies by other authors. In the meta-analyses that were conducted in China, age affected the decrease in the value of both physical health and psychological domains [20,21]. A study that was performed in Singapore, using the EQ-5D-5L, also demonstrated that older patients had a lower score and a poorer QoL [22].

The level of education among patients in our study exerted a significant influence on the parameters related to the environment domain of the WHOQOL-BREF. Specifically, higher levels of education were associated with lower total scores, indicating lower QoL for these patients. This finding suggests that higher education levels impact individuals’ perceptions and attitudes toward health and the various factors that influence their well-being [23]. Meta-analyses generally support the notion that a lower level of education is predictive of a poorer QoL among patients with schizophrenia [24,25]. In a study that was conducted in Singapore, the level of education demonstrated distinct correlations depending on gender. Among men, having primary and secondary school education was positively correlated with the social relationships domain, while among women, higher education levels were negatively correlated with both the social relationships and environment domains [26]. Research has consistently shown that the level of education significantly affects the QoL of these patients because it has a direct impact on intelligence, cognition, and brain development [27].

Regarding the number of patients in the room and the type of accommodation, specifically, whether they are four-bed, five-bed, or six-bed rooms, our research revealed that the environment domain score is directly influenced by the number of users in the room. This finding indicates that a higher number of users in the room has a positive impact on the QoL of these patients. This outcome aligns with expectations, as the QoL outcomes, including well-being, living conditions, and social functioning, are known to be influenced by factors such as social support, assistance, and engagement in various social activities. These factors are often facilitated within social protection institutions, as evidenced by similar studies [28,29].

The type of pavilion in which the patients were accommodated had a significant impact on the QoL of our patients, as well as on the scores on the BPRS and the physical health domain of the WHOQOL-BREF. Consequently, the scores on the mentioned scales reflected lower QoL for patients residing in the third pavilion. This outcome was expected, as the third pavilion primarily accommodates patients with more pronounced drug side effects, more severe psychiatric symptoms, and poorer overall health conditions.

The score of the social relationships domain of the WHOQOL-BREF in this study was found to be influenced by the age of onset of the disease. Specifically, a later onset of the disease was associated with lower scores and poorer QoL. This impact was particularly evident in questions related to satisfaction with one’s own qualities, sex life, and support from friends. Understanding the specific risk factors associated with different age groups can contribute to a better understanding of the etiology of the disease and help in the development of targeted services to meet the unique needs of each age group. In a study that was carried out in Melbourne, Australia, disease onset at a later age was associated with better early psychosocial functioning [30]. However, in women with a later disease onset, there were more associated comorbidities. On the other hand, if schizophrenia was diagnosed at an earlier age, another study showed that such patients were at a higher risk of unemployment, lower educational attainment (secondary or higher education), and a higher likelihood of living alone [31].

In our study, we found that the length of patients’ stay in the inpatient facility had an impact on the scores of the Q-LES-Q-SF scale, as well as the social relationships and environment domains of the WHOQOL-BREF. The longer the patients stayed in this institution, the lower the score was, indicating poorer QoL. There are various reasons for the prolonged stay of patients in such institutions, including clinical factors such as severe negative symptoms, inability to take care of themselves, the need for daily medical assistance, or violent behavior. Non-clinical factors such as issues with health insurance, and lack of family and community support may also contribute to the extended stay. In a study that took place in Ontario and systematic data were collected through the Ontario Mental Health Reporting System, it is stated that prolonged stay in psychiatric institutions led to an improvement in functional ability, which does not coincide with our research [32]. A study of a similar design indicates the importance of professional rehabilitation of these patients in case of a long stay in a psychiatric institution [33].

The score of the social relationships domain of the WHOQOL-BREF was influenced by the number of prescribed antipsychotics and the duration of therapy with a particular antipsychotic. Patients who were prescribed a higher number of antipsychotics per day had lower scores, indicating poorer QoL. On the other hand, patients who had been receiving antipsychotic therapy for a longer duration had higher scores, indicating improved QoL. The findings regarding the impact of polypharmacy on the QoL of patients with schizophrenia are consistent with other studies, which have shown that the use of multiple antipsychotics is associated with more severe negative symptoms and a higher frequency of side effects [34]. Furthermore, in line with similar studies, our results suggest that longer-term use of antipsychotics is associated with a lower mortality rate, prevention of relapse, and better social functioning in patients with schizophrenia [35,36]. It is important to note that a study conducted in Ethiopia using the same scale reported different results, indicating a negative correlation between the duration of treatment with a specific drug and the psychological domain, environment domain, social relationships domain, and overall QoL [37].

This research also highlights the impact of the number of daily doses of specific antipsychotics on the QoL of patients with schizophrenia. A higher number of daily doses of certain antipsychotics was associated with a decrease in the score for the environment and QoL domains. However, a higher number of daily doses of antipsychotics was also associated with better symptom control of schizophrenia, resulting in lower scores on the BPRS and improved QoL in this aspect. Existing literature supports a positive correlation between more frequent dosing of antipsychotics and the frequency of adverse reactions, as well as lower patient adherence [38]. Therefore, when determining the dosage regimen of antipsychotics, it is essential to carefully evaluate the benefit-to-risk ratio for each individual patient, taking into account the reduction of psychiatric symptoms and the intensity and clinical significance of adverse reactions [39].

Although assessing the QoL of patients in a research context is challenging, particularly for those with mental illnesses, it is necessary to consider not only the impact of medical care and treatment but also to evaluate individual perceptions of their personal circumstances in relation to cultural values, general norms, and their own goals, expectations, standards, and concerns. The literature provides evidence supporting the reliability of statistics obtained from individuals with schizophrenia. The results derived from the correlation analysis of all the aforementioned scales used in this study demonstrate their consistency in depicting the QoL of individuals with schizophrenia.

The main limitations in our study refer to the following facts. This study was performed only in one social welfare institution which could contribute to bias due to local treatment policies and varying availability of drugs. The limitations of our study encompass the relatively small number of patients. Due to the cross-sectional design of the study, we were unable to observe the influence of previous therapeutic protocols and metabolic changes associated with the use of atypical antipsychotics. Also, the caregivers of these patients were not included in the research.

Results of this research indicate poorer QoL in patients with schizophrenia where age, level of education, type of accommodation, type of pavilion, age of onset of the disease, number of prescribed antipsychotics, number of psychiatric comorbidities, duration of therapy, and the number of daily doses of antipsychotics are foremost predictors of poor QoL in patients with schizophrenia which are treated in inpatient institution. In summary, the results from this study using cross-culturally valid questionnaires and a wide range of predictors could underline the medical, psychological, and social burden that schizophrenia puts on society and make a basis for planning future improvement in health care aimed to achieve the highest possible QoL in these patients.
